# T3 therapy in hypothyroidism. Still more questions than answers

**DOI:** 10.20945/2359-3997000000378

**Published:** 2021-06-29

**Authors:** Marcio José Concepción Zavaleta, Julia Cristina Coronado Arroyo, Francisca Elena Zavaleta Gutiérrez, Luis Alberto Concepción Urteaga

**Affiliations:** 1 Hospital Nacional Guillermo Almenara Irigoyen División de Endocrinología Lima Perú División de Endocrinología, Hospital Nacional Guillermo Almenara Irigoyen, Lima, Perú.; 2 Clínica Vesalio División de Obstetricia y Ginecología Lima Perú División de Obstetricia y Ginecología, Clínica Vesalio, Lima, Perú.; 3 Hospital Belén de Trujillo División de Neonatología La Libertad Perú División de Neonatología, Hospital Belén de Trujillo, Trujillo, La Libertad, Perú.; 4 Hospital Regional Docente de Trujillo División de Neumología La Libertad Perú División de Neumología, Hospital Regional Docente de Trujillo, Trujillo, La Libertad, Perú.

**DEAR EDITOR,**

We have read with interest the article published by Dora J, which recommends against prescribing liothyronine (L-T3) alone or in combination with levothyroxine (LT4) for hypothyroidism, because the short half-life of triiodothyronine (T3) means multiple daily doses are required, and there is no clear evidence in humans that combination therapy is superior to L-T4 alone (1). No guidelines have recommended thyroid hormone replacement with L-T3 alone, but the question remains whether L-T3 + L-T4 combination therapy may be better than levothyroxine alone in a selected group of patients with hypothyroidism (2).

Deiodinases are enzymes that mediate the activation and inactivation of thyroid hormones. Genetic variation in these enzymes is associated with altered thyroid function and adverse health outcomes. Studies have shown that individuals with genetic variations in deiodinase type 2 and the thyroid hormone transporter protein MCT10 experience additional benefits with combined LT3 + LT4 therapy, though the sample size was small (3). On the other hand, single nucleotide polymorphisms may have little discriminatory power to determine which patients could benefit from combination therapy (3).

The 2012 ETA guideline suggests that combined therapy could be considered experimentally in hypothyroid patients treated with L-T4, who have persistent complaints despite having target TSH values, provided they have previously given support to deal with the chronic nature of their disease and associated autoimmune diseases have been ruled out (4). The NICE guideline does not give a clear recommendation on the use of liothyronine, but states that, although long-term safety is uncertain, it may play a role in patients with symptoms of hypothyroidism despite adequate replacement with levothyroxine (5).

Recently, the 2021 ETA Consensus established established evidence-based recommendations and patient criteria for LT3 + LT4 combination therapy in hypothyroidism. After excluding other causes of persistent symptoms, patients who do not report clinical improvement with a dose of at least 1.2 ug/kg/day of levothyroxine should be considered for combination therapy. Likewise, those with low baseline serum total T3 levels while taking LT4 monotherapy should also be include. Noteworthy, they suggest that future combination therapy trials should consider including polymorphism genotyping, and should be adequately powered to study the effect of this polymorphism on trial outcomes (6).

The recommended initial dose of LT4:LT3 is 13:1 to 20:1, a ratio that mimics the ratio of physiological thyroxine (T4) and T3 secretion by the human thyroid gland, representing a dose of 5 or 10 μg LT3 for patients taking 100 to 200 μg LT4. If no improvement is seen after 3-6 months of treatment, assessed by questionnaires such as ThyPRO, treatment should be discontinued (6).

We emphasize that more studies are needed to identify the subgroup of hypothyroid patients that may benefit from the use of liothyronine, in addition to levothyroxine, probably through the identification of new biomarkers or genetic polymorphisms.
